# Identification of a novel candidate HSD3B2 gene variant for familial hypospadias by whole-exome sequencing

**DOI:** 10.3389/fgene.2023.1106933

**Published:** 2023-06-13

**Authors:** Hamdi Hameed Almaramhy, Firoz Abdul Samad, Ghadeer Al-Harbi, Dimah Zaytuni, Syed Nazar Imam, Tariq Masoodi, Monis Bilal Shamsi

**Affiliations:** ^1^ College of Medicine, Taibah University, Medina, Saudi Arabia; ^2^ College of Applied Medical Science, Taibah University, Medina, Saudi Arabia; ^3^ Centre for Genetics and Inherited Diseases, Taibah University, Medina, Saudi Arabia; ^4^ Translational Medicine Department, Research Branch, Sidra Medicine, Doha, Qatar; ^5^ Department of Biochemistry, College of Medicine, Taibah University, Medina, Saudi Arabia

**Keywords:** hypospadias, 3β-hydroxysteroid dehydrogenase 2, genetic risk factors, whole-exome sequencing, Y-chromosome microdeletion

## Abstract

**Introduction:** Hypospadias [MIM: 300633] is one of the most frequent congenital malformations of male external genitalia. The spectrum of genetic variants causing hypospadias is varied, with studies commonly implicating genes critical in the fetal steroidogenic pathway. This is the first genetic study on hypospadias from the Yemen ethnicity and the second to report *HSD3B2* mutations in more than one affected individual from the same family.

**Material and methods:** Surgical hypospadias repair was performed on two hypospadias-affected siblings from a consanguineous family. Whole-exome sequencing (WES) was performed to identify the potential pathogenic variant for hypospadias, which was later confirmed by Sanger sequencing. The identified variant was further analyzed for its pathogenicity by using *in silico* tools such as SIFT, PolyPhen-2, MutationAssessor, MutationTaster, FATHMM, and ConSurf.

**Results:** We identified a novel missense mutation (Chr1:119964631T>A, c.507T>A, p. N169K) in 3β-hydroxysteroid 2-dehydrogenase (*HSD3B2*) gene by WES. Sanger sequencing confirmed that the variant segregated the disease in the family between the affected and non-affected individuals. Both patients are homozygous, while parents and two unaffected siblings are heterozygous carriers, indicating an autosomal recessive pattern of inheritance. The *in silico* analysis by all six *in silico* tools (SIFT, PolyPhen-2, MutationAssessor, MutationTaster, FATHMM, and ConSurf) predicted the variant to be pathogenic/deleterious.

**Discussion:** An abnormal fetal steroidogenic pathway due to genetic influences may affect the development of the male genital tract, including the urethral tract closure and morphogenesis of male genitalia. Furthermore, the pathogenicity of the observed variant in this study, confirmed by multiple *in silico* tools, characterizes the influence HSD3B2 gene variants may have in the etiology of hypospadias.

**Conclusion:** Understanding of pathogenic manifestation and inheritance of confounding genetic variants in hypospadias is a matter of great concern, especially in familial cases.

## Introduction

Hypospadias is congenital hypoplasia of the penis, leading to a ventrally displaced urinary meatus, often associated with a dorsal-headed foreskin and chordee (Leung and Robson, 2007). It is the second most common male urogenital abnormality after cryptorchidism (Ságodi et al., 2014). The prevalence of hypospadias varies widely between different populations (0.26–47 per 10,000 births), with reports suggesting higher frequency in white people than in black people and Asians (KŠllŽn et al., 1986; Paulozzi, 1999; Kurahashi et al., 2004; Yang et al., 2004; Carmichael et al., 2007).

Hypospadias is characterized by incomplete fusion of the urethral folds during early embryonic development (Baskin and Ebbers, 2006)*.* From the 8th week onwards, when gonads have differentiated into testes in XY embryonic males, the external genitalia development and urethral modifications are mainly dependent on the secretion and action of androgens (Baskin and Ebbers, 2006; Bouty et al., 2015), in particular, by testosterone produced from the fetal testes and transformed peripherally into dihydrotestosterone that binds to the androgen receptors. The cascades of hormonal activities influenced by numerous genetic factors induce fusion of the urethral folds along the ventral surface of the penis and produce a series of morphological changes in the developing external genitalia (Baskin and Ebbers, 2006). During this hypospadias-critical gestational window (8–14 weeks), the synthesis, concentration, timing, and metabolism of hormones/androgens are important for proper urethral closing and external genitalia development, and any abnormality may result in hypospadias (Bouty et al., 2015)*.*

Numerous lines of evidence suggest a genetic component as an important etiology, with the presence of an affected family member being a high risk factor (van der Zanden et al., 2012; Thorup et al., 2014)*.* First-, second-, or third-degree relatives are affected in 7% of patients with familial clustering of hypospadias (Fredell et al., 2002)*.* In most familial cases, hypospadias shows equal transmission through both maternal and paternal lines with similar recurrence risks between the brothers and sons of patients (Schnack et al., 2008)*.* The chances of hypospadias increase by 9%–17% for the brother of a male with hypospadias. Pedigree and twin studies have reported a higher genetic component of 57%–77% in the heritability of hypospadias (Schnack et al., 2008).

The risk of congenital abnormalities is higher in children born from consanguineous parents. Investigating such affected families provides a higher prospect of genetic findings. Therefore, we investigated a consanguineous family with two hypospadias-affected siblings using whole-exome sequencing. The bioinformatics tools for *in silico* predictions were applied to assess the structural and functional consequences of the variants observed in the study.

## Subjects and methods

### Patients

The patients (two male siblings) were brought to the Saudi German Hospital in Medina, Saudi Arabia, for surgical correction of hypospadias. The family also had two non-affected (normal) male siblings. The family was ethnically from Yemen, and the parents were consanguineous. The affected siblings responded well to the treatment, and no complications during or pre/post-surgery were observed. The Institute’s ethical committee approved the study, and written informed consent was taken from study participants.

#### Whole-exome sequencing (WES)

**FIGURE 1:**
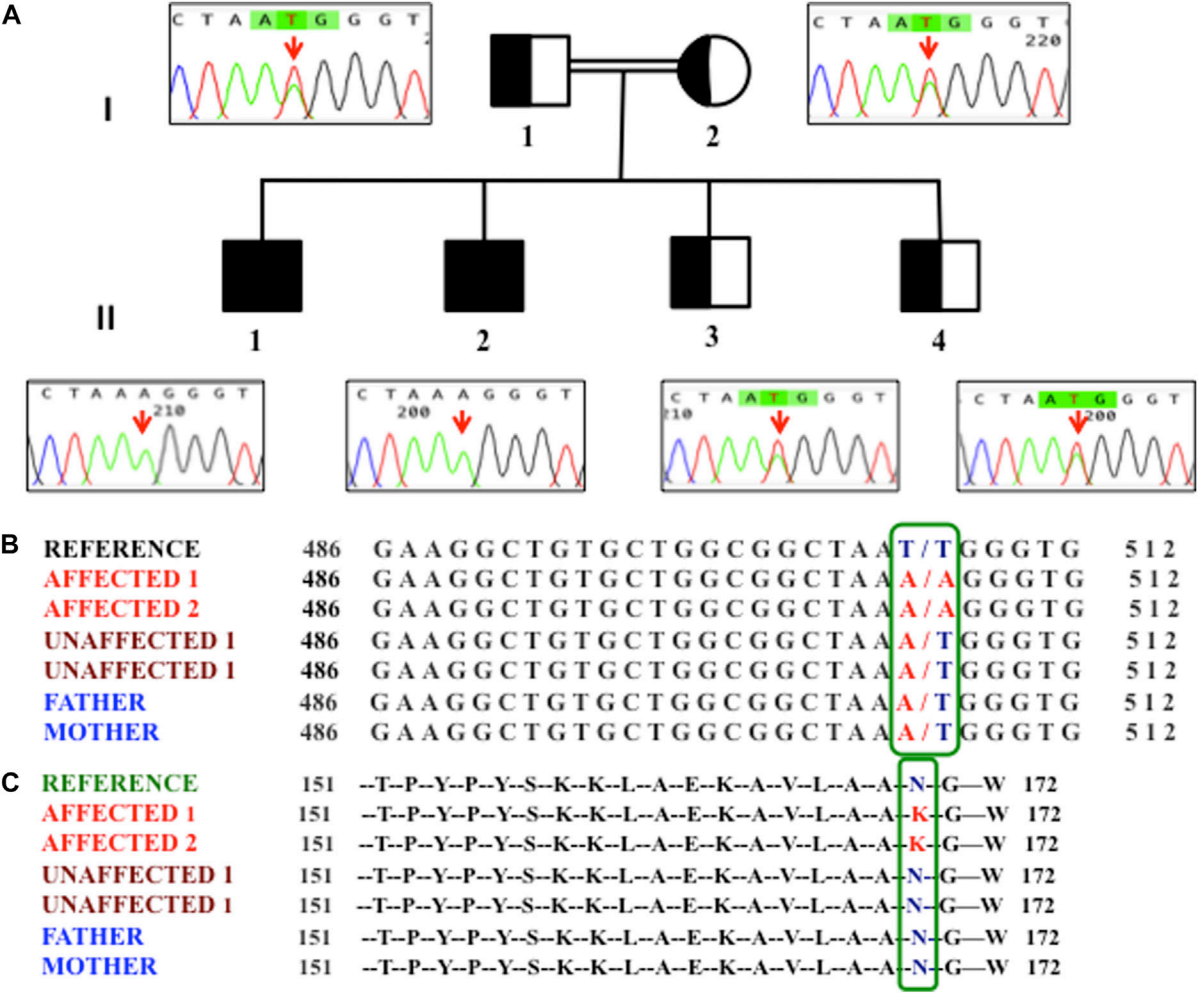
**(A)** Pedigree of the family showing homozygosity (II1 and II2) and hetrozygosity (I1, I2, II3, and II4). An arrow in the chromatogram highlights the T507A variant. **(B)** Nucleotide variants observed at the c.507 position of the HSD3B2 gene in members of the family. **(C)** Amino acid variant observed at the 169th position in members of the family.

Whole-exome sequencing (WES) was performed on venous blood DNA from the parents and three siblings (two affected and one normal child) as per the manufacturer’s instructions (Exome RDY Kit Cat. No: A38262, Thermo Fisher Scientific, USA). Sequence reads were aligned to a reference genome (hg19) (http://genome.ucsc.edu/), and variants were identified using the Ion Torrent pipeline (Thermo Fisher Scientific, USA). Variants that show an effect on the protein sequence (missense, nonsense, frameshift, splicing, etc.) and variants with minor allele frequency ≤0.05 in the 1000 Genomes Project (ftp://ftp.1000genomes.ebi.ac.uk/vol1/ftp) were considered in downstream analysis. We then identified the suspected pathogenic variants shared between the affected children by comparing each gene variant among the family members. The identified variant was further validated by Sanger sequencing among all the family members (both parents, two affected, and two non-affected siblings) (Figure 1). The primers (Supplementary Table S1) were designed using Primer3 software (https://primer3.ut.ee/).

### Yq microdeletion analysis

Y-chromosome microdeletions were analyzed as described by Shamsi et al. (2012). Briefly, multiplex analysis using STS primer sets, which spanned the AZFa, AZFb, and AZFc regions of the Y chromosome for sY84, sY86 (AZFa); sY127, sY134 (AZFb); and sY254, sY255 (AZFc) was performed on genomic blood DNA of hypospadias-affected and non-affected siblings. sY14 (SRY) was used as an internal control, while a male DNA and a female DNA sample were used as positive and negative controls, respectively.

### Computational analysis for pathogenicity prediction

We used six different *in silico* tools to predict the pathogenicity potential of the variant. Each *in silico* tool predicted the pathogenicity potential based on distinct criteria and parameters. The tools used for pathogenicity prediction are SIFT (https://sift.bii.a-star.edu.sg/), PolyPhen-2 (http://genetics.bwh.harvard.edu/pph2/), MutationTaster (http://www.mutationtaster.org/), MutationAssessor (http://mutationassessor.org/r3/), FATHMM (http://fathmm.biocompute.org.uk/index.html), and ConSurf Analysis (https://www.bio-sof.com/consurf) (https://consurf.tau.ac.il/).

## Results

### Clinical presentation and family history

The eldest two sons, aged 11 and 9 years, had hypospadias, while the younger two sons (6 and 4 years) were normal on clinical examination. At the time of sample collection, the father was 38 years old and the mother was 27 years old. The meatal position was proximal (penoscrotal), and the penile chordee was present in both the affected siblings. The testes were descended and normal. We did not observe Y-chromosome microdeletions in the hypospadias-affected or non-affected siblings. No medical history of hypospadias or any other genetic disorder or congenital malformation is reported in the family.

### Genomic analysis

We performed WES in two hypospadias-affected siblings, one unaffected sibling, and both parents. A total of 6,072 exonic variants were observed; the majority were found to be missense (5,541 variants). We also observed 172 variants as homozygous, where both alleles were altered (Supplementary Table S2). After annotation and filtering, we observed a novel variant T507A in the 3β-hydroxysteroid 2-dehydrogenase (*HSD3B2*) gene, which segregated the disease in the family between the affected and non-affected individuals. Furthermore, we validated the T507A variant by Sanger sequencing among all members of the family, including one unaffected sibling who was not investigated by WES. We observed that the other unaffected sibling was also heterozygous for the same variant (Figure 1). We checked this T507A variant in an additional age- and gender-matched control cohort containing whole-exome sequencing of 127 males, and, interestingly, this variant was absent (0/127) in all control individuals. We also screened for this variant in the Greater Middle East (GME) Variome database (http://igm.ucsd.edu/gme/index.php), which has WES data for 2,497 healthy individuals (controls) as a genomic reference of the Middle Eastern population, and could not find any prior occurrence of our variant in their cohort. Additionally, we also searched in the Genome Aggregation Database (gnomAD), which has whole-exome and whole-genome data on 125,748 and 15,708 individuals, respectively, and our variant did not match with any of the early reported variants. No Yq microdeletion was observed in the affected or non-affected male siblings.

*HSD3B2* is related to the etiology of hypospadias and was reported as causative or contributory to hypospadias in earlier studies (Codner et al., 2004a; Wang et al., 2007; Rabbani et al., 2012) due to its role in the synthesis of endocrinal factors that regulate fetal urogenital system development (van der Zanden et al., 2012). However, the T507A variant found in our study has not yet been reported. We confirmed the novelty of our T507A variant by a search in previous publications, various databases (ftp://ftp.1000genomes.ebi.ac.uk/vol1/ftp), (https://www.ncbi.nlm.nih.gov/snp/), and particularly in the Middle-East-specific database (http://igm.ucsd.edu/gme/data-browser.php) due to the ethnically Yemen origin of our study subjects.

The *HSD3B2* gene has four exons and three introns, and the candidate variant T507A is located in exon 4 of the *HSD3B2* gene. The non-synonymous T507A variant caused an amino acid change at the 169th position from asparagine (N) to lysine (K). The T507A variant in the *HSD3B2* gene is located in the evolutionarily conserved region of the genome among the closely related primates, such as chimpanzees, gorillas, and macaques, which represents its functional significance (Table 1).

**TABLE 1:** Comparison of evolutionarily conserved regions corresponding to the N169K change of HSD3B2 observed in our patients with closely related primates, such as chimpanzees, gorillas, and macaques.

Patient 1 and 2	160 -L--A--E--K--A--V--L--A--A--K--G--W--N--L--K--N--G--D--T- 178
Human (*Homo sapiens*)	160 -L--A--E--K--A--V--L--A--A--N--G--W--N--L--K--N--G--D--T- 178
Chimpanzee (*Pan troglodytes*)	160 -L--A--E--K--A--V--L--A--A--N--G--W--N--L--K--N--G--D--T- 178
Gorilla (*Gorilla gorilla*)	160 -L--A--E--K--A--V--L--A--A--N--G--W--N--L--K--N--G--D--T- 178
Macaque (*Macaca mulatta*)	161 -L--A--E--K--A--V--L--A--A--N--G--W--T--L--K--N--G--G--T- 179

### Pathogenicity prediction

The *in silico* pathogenicity predictions used in this study were based on a combination of nucleotide/amino acid characteristics as primary DNA sequence features (PolyPhen-2**)**, biochemical properties of substituted/variant amino acid (SIFT), protein structural features (PolyPhen-2), homology evolutionary conservation (SIFT, PolyPhen-2, MutationAssessor, FATHMM, and ConSurf Analysis), association/susceptibility to disease (MutationTaster and FATHMM), and the presence/proximity of the variant to the substrate binding/catalytic site (ConSurf Analysis) (Adzhubei et al., 2010; Schwarz et al., 2010; Sim et al., 2012; Carter et al., 2013; Shihab et al., 2013; Kircher et al., 2014; Quang et al., 2015; Rogers et al., 2018) (https://sift.bii.a-star.edu.sg/; https://ionreporter.thermofisher.com/ionreporter/help/GUID-57A60D00-0654-4F80-A8F9-F6B6A48D0278.html; http://www.mutationtaster.org/info/documentation.html; https://omictools.com/mutationassessor-tool; http://fathmm.biocompute.org.uk/inherited.html, http://fathmm.biocompute.org.uk/index.html; http://consurf.tau.ac.il/gallery.php). The *in silico* analysis of the six applied tools concordantly predicted the T507A variant as potentially deleterious considering the structural, functional, disease susceptibility, phylogenetic, and evolutionary aspects of the substitution (Table 2).

**TABLE 2:** Computational analysis for pathogenicity prediction of the T507A variant by *in silico* tools (SIFT, PolyPhen-2, MutationTaster, MutationAssessor, FATHMM, and ConSurf Analysis).

S. No.	*In silico* tool	Criteria	T507A variant score	Prediction
1	SIFT	Interprets protein function loss based on the sequence homology comparison of evolutionarily conserved regions	0	Deleterious to protein function
2	PolyPhen-2	Analyzes structural and function damage based on the sequence, and phylogenetic and structural parameters	0.886	Pathogenic to protein structure and function
3	MutationTaster	Evaluates disease-causing potential based on evolutionary conservation, splice site changes, protein features, and mRNA characteristic alterations	0.993	Damaging to protein function and pathogenic
4	MutationAssessor	Interprets functional damage to protein based on evolutionary conservation patterns in protein family multiple sequence alignments	3.595	Highly damaging to protein function
5	FATHMM	Uses the hidden Markov modeling approach to calculate position-specific probabilities and evaluate functional impact	−2.6	Damaging to the function
6	ConSurf Analysis	Reveals evolutionary conservation of nucleic acid positions in a DNA molecule based on the phylogenetic relations between homologous sequences	7	Damaging to the function

## Discussion

The identified novel homozygous variant (T507A) in HSD3B2 genetically segregates the affected and non-affected individuals of the family. Both the parents and the two non-affected male siblings were heterozygous carriers (T/A), suggesting an autosomal recessive pattern of inheritance.

3β-Hydroxysteroid 2-dehydrogenase (3β-HSD2), a product of the *HSD3B2* gene and an isozyme of 3β-hydroxysteroid dehydrogenase (3βHSD), is important for the biosynthesis of steroids, in particular, testosterone (Morrison et al., 1991; Bain et al., 1993). It is expressed predominantly in the adrenal gland, the ovary, and the testis (Lachance et al., 1992). Its role and function are implicated in several studies on hypospadias (Codner et al., 2004a; Carmichael et al., 2014; Kalfa et al., 2019)**.** In embryonic males, the enzymatic steps that contribute to steroidogenesis mainly occur in the fetal Leydig cells *via* the steroidogenic pathway, which converts cholesterol into sex hormones, especially testosterone and its derivative dihydrotestosterone. The product of *HSD3B2* is involved in enzymatic conversion of numerous precursors, such as pregnenolone to progesterone, 17αOH pregnenolone to 17αOH progesterone, dehydroepiandrosterone (DHEA) to androstenedione, and androstenedione to testosterone (Carmichael et al., 2014; Bouty et al., 2015)*.* These are critical for the hormone-dependent development of the male genital tract, including the urethral tract closure and morphogenesis of male genitalia. Abnormalities in this development cause hypospadias (Kotula-Balak et al., 2012)*.* Physiologically, the T507A variant observed in our probands may have affected the normal fetal steroidogenic pathway, leading to hypospadias in the affected siblings.

Numerous loci variants for *HSD3B2* are reported in earlier studies (Codner et al., 2004a; Wang et al., 2007; Rabbani et al., 2012; Kon et al., 2015). However, the T507A variant (N169K amino acid change) observed in our study is novel and not reported earlier. A ClinVar database (https://www.ncbi.nlm.nih.gov/clinvar/) search for the HSD3B2 variant suggests that most genetic changes in HSD3B2 are associated with abnormalities in the urogenital system (Supplementary Table S3). In a systematic mutation screening of 25 known causative/candidate/susceptible genes in Japanese and Vietnamese patients, Kon M et al. suggested that *HSD3B2* mutations may potentially lead to non-syndromic hypospadias as a sole clinical manifestation (Kon et al., 2015)*.* In another study, *HSD3B2* was genotyped in 90 hypospadias patients and 101 healthy controls. Two missense heterozygous mutations (S213T and S284R) and three heterozygous variants (A238, T259, and T320) with no amino acid change were observed in patients but not in controls. These findings suggested that subtle molecular abnormalities in the *HSD3B2* gene may be observed in idiopathic hypospadias patients (Codner et al., 2004a)*.* A 27-bp deletion (687del27) in exon IV, deleting the terminal base pair of codon 229 and the entire codons 230–237, in addition to the first two base pairs of codon 238, is also reported in a normozoospermic man with hypospadias (Donadille et al., 2018)*.* A homozygous missense mutation of A82P in exon 3 of the *HSD3B2* gene is also reported in a hypospadias patient (Rabbani et al., 2012)*.* Therefore, mutations of *HSD3B2* underlie various types of hypospadias, as shown by the evidence of these earlier studies.

**FIGURE 2:**
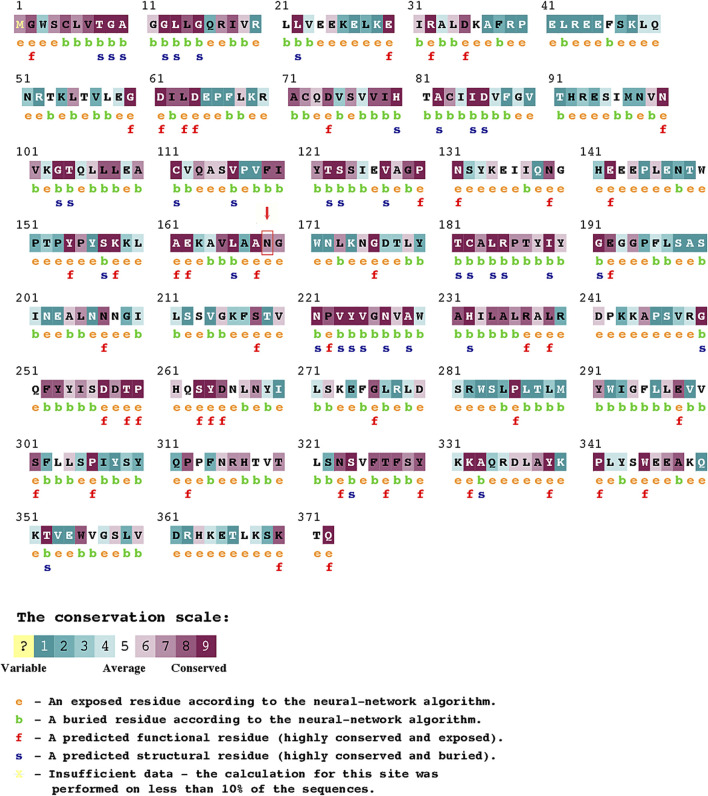
ConSurf Analysis predicting the pathogenicity score for variations in individual amino acids in HSD3B2.

In addition, *in silico* analysis (using SIFT, PolyPhen-2, MutationTaster, MutationAssessor, FATHMM, and ConSurf) consistently predicted the severely deleterious nature of the observed T507A variant in our patients. We employed six *in silico* tools to increase the stringency of our analysis because individual tools often disagree, in part because they use different predictive features. Together, these tools encompass a broad range of non-synonymous variant/amino acid classification criteria and methods, collectively making the final predicted outcome more accurate. Three important features that concern the mutant missense variant (N169K) observed in our study are as follows: 1) our variant is in close proximity to one of the two catalytic sites (amino acid 154–158 and 269–273) reported in earlier mutagenesis and structural studies (Morel et al., 1997; Thomas et al., 2002; Simard et al., 2005). The amino acid substitutions located at or near the binding interface or active site could hinder the active site, change the recognition, alter the specificity, or affect the binding affinity (Parker, 2001; Mata et al., 2006)*.* Therefore, the deleterious influence of amino acid substitution on HSD3B2 enzyme activity in our patients cannot be overruled (Figure 2). 2) The N169K variant is located in a locus that is evolutionarily conserved across closely related species (Table 1; Figure 2). The degree of evolutionary conservation of an amino acid reflects the structural and functional importance of the domain. The higher the evolutionary conservation, the more critical is the function of the domain/protein and the need to retain the domain/protein’s structural integrity (Ashkenazy et al., 2016)*.* **3)** The T507A mutation substituted the neutral amino acid asparagine with positively charged (basic) lysine.

Although endocrinal disorder has been reported in hypospadias, the percentage of patients with identified endocrinal etiology remains low (Kalfa et al., 2009). Cassorla et al., 2004 reported that isolated patients with hypospadias are more likely to have normal endocrine testicular and androgen end-organ functions (Codner et al., 2004b). In a study by Rey et al. (2005), of 61 patients, only 13 (21.31%) were found to have an androgen synthesis defect (Rey et al., 2005). As development of the urethra occurs during 8–16 weeks gestation, the postnatal hormonal milieu may not reflect early gestation events (Baskin, 2000; Baskin et al., 2001; Holmes et al., 2004). Unfortunately, we could not assess the hormone levels in our patients. However, any relatable clinical feature or symptom associated with any enzyme or hormone imbalance, in particular, adrenal insufficiency, was not observed in our patients.

Inconsistent findings have been reported for the presence of Y-chromosome microdeletions in hypospadias patients (Tateno et al., 2000; Juniarto et al., 2018). We did not observe Y chromosome microdeletions in any of the hypospadias-affected or non-affected siblings in our study.

To the best of our knowledge, this is the second reported familial hypospadias case with *HSD3B2* mutations in more than one affected individual from the same family (siblings). The previously published *HSD3B2* mutation in hypospadias siblings was published by Ladjouze et al. (2022). In addition, this is the first genetic study on hypospadias from the Yemen ethnicity. Interestingly, an ethnicity-specific difference in gene function is also reported for *HSD3B2* variants between Caucasian-American, African-American, Han Chinese-American, and Mexican-American populations (Wang et al., 2007).

In summation, we described a novel variant in the *HSD3B2* gene with an autosomal recessive inheritance that probably affected fetal androgen synthesis and caused hypospadias in two siblings. This clinical case re-emphasizes the importance of structurally and functionally intact HSD3B2 in proper urethral folding and external genitalia morphogenesis during fetal life. *In silico* analysis predicted the variant to be potentially pathogenic to the structure and function of the *HSD3B2* gene product. While we have unraveled a single locus for hypospadias, future genetic and functional studies, particularly in patients from the same parents, will provide a better understanding of disease etiology and inheritance. Additionally, it will aid in developing strategies for improved management of future pregnancies in high-risk familial cases.

## Data Availability

The datasets presented in this study can be found in online repositories. The names of the repository/repositories and accession number(s) can be found at: https://www.ncbi.nlm.nih.gov/, PRJNA982072 and https://www.ncbi.nlm.nih.gov/clinvar/docs/submit/, SUB12530918.

## Abbreviations

DHEA, dehydroepiandrotone; HSD3B2, 3β-hydroxysteroid 2-dehydrogenase; WES, whole-exome sequencing.
