# Supplementary material for: Identification of a novel candidate HSD3B2 gene variant for familial hypospadias by whole-exome sequencing
Source: Front Genet. 2023 Jun 13;14:1106933. doi: 10.3389/fgene.2023.1106933 (PMC10297146; doi:10.3389/fgene.2023.1106933)
Supplement: Supplementary file 2 [file Table1.docx]

Forward: ATGTGGTTGCAGCTCCTTTG

Reverse: AGGCGTGTCATCTGAGATGT

Product Size: 533bp
